# Multiple Mammalian Cytokines and Erythropoietin‐Mimetic Peptides Protect Insect Neurons via Phylogenetically Conserved Cytokine Receptor‐Like Factor 3 (CRLF3)

**DOI:** 10.1111/jnc.70207

**Published:** 2025-09-03

**Authors:** Nina Hahn, Debbra Y. Knorr, Björn Twellsieck, Ruoyu Huang, Abigail Trebilcock, Nicola Schwedhelm‐Domeyer, Stephanie Pauls, Lars v. Werven, Olaf Jahn, Hannelore Ehrenreich, Ralf Heinrich

**Affiliations:** ^1^ Section of Translational Neuroimmunology, Department of Neurology and Center for Sepsis Control and Care Jena University Hospital Jena Germany; ^2^ Basic & Clinical Neuroscience Department King's College London London UK; ^3^ Cellular Neurobiology, Institute for Zoology and Anthropology Georg‐August‐University Göttingen Göttingen Germany; ^4^ Werner Reichardt Centre for Integrative Neuroscience, Institute of Neurobiology University of Tübingen Tübingen Germany; ^5^ Neuroproteomics Group, Department of Molecular Neurobiology Max‐Planck‐Institute for Multidisciplinary Sciences Göttingen Germany; ^6^ Translational Neuroproteomics Group, Department of Psychiatry and Psychotherapy University Medical Center Göttingen Göttingen Germany; ^7^ Experimental Medicine, Central Institute of Mental Health, Medical Faculty Mannheim Heidelberg University Heidelberg Germany; ^8^ Clinical Neuroscience Max‐Planck‐Institute for Experimental Medicine Göttingen Germany

**Keywords:** cytokine receptor‐like factor 3 (CRLF3), erythropoietin, erythropoietin‐mimetic peptides, evolution, human cytokines, neuroprotection

## Abstract

Erythropoietin (Epo) and its non‐erythropoietic splice variant EV‐3 have demonstrated potent neuroprotective effects across species, although the respective mechanisms are variable and incompletely understood. Unlike vertebrates, insects lack both Epo and the classical Epo receptor but express Cytokine Receptor‐Like Factor 3 (CRLF3), a conserved type I receptor that serves as a neuroprotective receptor for Epo and EV‐3 in insects and human iPSC‐derived neuron‐like cells. Insects, which express CRLF3 but lack all other group 1 type I cytokine receptors, represent a suitable model to study the function of CRLF3 in neuroprotection. Previous experiments with primary brain neurons from the locust *Locusta migratoria* demonstrated that human Epo and EV‐3 prevented hypoxia‐induced apoptosis via activation of CRLF3 and downstream signaling mediated by JAK/STAT activity. Here we demonstrate that locust neurons are also protected by three small peptides (≤ 20 amino acids) with previously established erythropoietic and/or neuroprotective capacities in mammals. These peptides included non‐erythropoietic HBSP and P16, as well as erythropoietic EMP1. To further characterize the ligand spectrum of locust CRLF3, we tested specific ligands of vertebrate group 1 type I cytokine receptors for their neuroprotective potential. While human thrombopoietin protected locust neurons via activation of CRLF3, human prolactin and growth hormone showed no anti‐apoptotic effects. Our findings highlight CRLF3 as a versatile neuroprotective receptor that can be activated by the three natural human cytokines Epo, EV‐3, and thrombopoietin, and three artificial short peptides with and without erythropoietic activity. In contrast to vertebrate classical Epo receptor, which is only activated by Epo and EMP1, CRLF3 has an extended and more generalist ligand spectrum to mediate neuroprotection. This wide ligand spectrum may support the hypothesis of CRLF3 being the evolutionary precursor of various vertebrate‐specific cytokine receptors and may qualify this more general cell protective receptor as a target for pharmacological intervention in neurodegenerative processes.

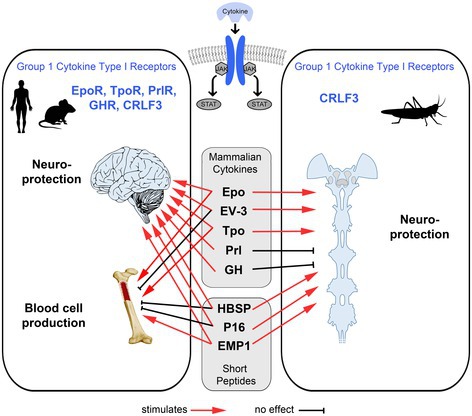

## Graphical abstract

Mammals express five different structurally related cytokine type I receptors: erythropoietin‐receptor, thrombopoietin‐receptor, prolactin‐receptor, growth hormone‐receptor and cytokine receptor‐like factor 3 (CRLF3). Of these, only CRLF3 exists in insects and other eumetazoan invertebrates. Insect CRLF3 mediates neuroprotection following activation with vertebrate‐specific cytokines Epo, EV‐3, and Tpo but not Prl and GH. Insect CRLF3 is also activated by short peptides HBSP, P16 and EMP1 that also stimulate neuroprotection and/or blood cell production in mammals. The generalist ligand spectrum of insect CRLF3 serving cell protection may support the hypothesis of CRLF3 being the evolutionary precursor of various cytokine receptors with specific ligands mediating specific functions in different vertebrate tissues.

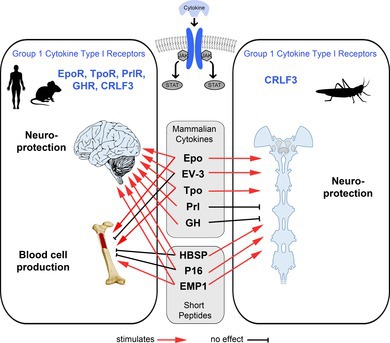

**Abbreviations**
CRLF3: cytokine receptor‐like factor 3
DAPI: 4′,6‐diamidino‐2‐phenylindole, dihydrochloride
dsRNA: double‐stranded ribonucleic acid
EMP1: synthetic peptide not related to Epo primary sequence
Epo: erythropoietin
EpoR_2_
: homodimeric (classical) erythropoietin receptor
EV‐3: Epo splice variant lacking amino acids encoded by the third exon
FCS: fetal calf serum
G‐CSF: granulocyte‐colony stimulating factor
GH: growth hormone
GHR: growth hormone receptor
GM‐CSF: granulocyte macrophage‐colony stimulating factor
H/h: Hour/h
HBSP: Epo B helix‐derived helix B surface peptide
HPLC: high pressure liquid chromatography
JAK: janus kinase
P16: Epo A helix‐derived peptide
PBS: phosphate‐buffered saline
PBST: phosphate‐buffered saline with Triton‐X‐100
Prl: prolactin
PrlR: prolactin receptor
RNA_i_
: RNA interference
STAT: signal transducer and activator of transcription
Tpo: thrombopoietin
TpoR: thrombopoietin receptor
β‐cR: beta common chain receptor (can form heteromeric receptor with EpoR)

## 1 Introduction

Cytokines and their receptors regulate immune functions, inflammatory processes, proliferation, cell differentiation, maturation, and regeneration in animals across phyla (Liongue, Sertori, and Ward 2016; Dinarello 2007). They belong to several distinct families characterized by sequence similarities and shared structural components. Type I cytokine receptors contain a characteristic extracellular cytokine receptor homology domain (CHD) and a membrane proximal W‐S‐X‐W‐S amino acid motive. Functional receptors form homo‐ or heteromeric complexes that initiate cellular responses via janus kinase (JAK) activity (Boulay et al. 2003; Liongue, Sertori, and Ward 2016). Corresponding type I cytokines have been reported to exist in all vertebrate species. They share little primary sequence similarity but contain a bundle of four α‐helices as a common structural feature (Boulay et al. 2003; Huising et al. 2006; Liongue and Ward 2007; Ghezzi and Conklin 2013). Cytokine signaling elicits pleiotropic effects and is versatile with respect to receptors activated by a particular cytokine, multiple cytokines activating the same receptor, and functional receptor complexes being composed of different combinations of homo‐ or heteromeric partners (Ozaki and Leonard 2002; Shing et al. 2018; Chauhan et al. 2021; McFarlane et al. 2021).

While rather few cytokines and cytokine receptors have been identified in invertebrates, vertebrates evolved numerous cytokines and corresponding receptors that regulate cell type‐specific functions in particular tissues (Beschin et al. 2001; Ocampo et al. 2009). One example is the production and differentiation of certain blood and immune cells by erythropoietin (Epo–erythrocytes), thrombopoietin (Tpo–thrombocytes), granulocyte‐colony stimulating factor (G‐CSF–neutrophil white blood cells), granulocyte macrophage‐colony stimulating factor (GM‐CSF–granulocytes and macrophages) and others (Metcalf 2008). Remarkably, these cytokines and their receptors are also expressed in non‐hematopoietic tissues including the central nervous system, where they typically reduce apoptotic cell death and promote survival and regeneration of compromised cells (Byts et al. 2006; Tönges et al. 2007; Ghezzi and Conklin 2013). The origin of partially shared and partially specific functions of these cytokines and their receptors is currently not clear. In this regard, Ghezzi and Conklin (2013) hypothesized that general tissue‐protective activities of many helical cytokines preceded their specific functions on particular cell types that developed later when blood cells and immune systems evolved in vertebrate lineages. Furthermore, it was suggested that early vertebrates expressed one ancestral cytokine receptor gene, which, following gene duplication events (1R and 2R), gave rise to more specific receptors found in extant vertebrates (Huising et al. 2006; Ocampo et al. 2009). Liongue, Taznin, and Ward (2016) specifically suggested Cytokine Receptor‐like Factor 3 (CRLF3) as the potential evolutionary precursor of all cytokine receptors.

CRLF3 (synonyms: CYTOR4, CREME9, p48.2, p48.6) possesses various typical features of cytokine receptors, but its manifold functions in different tissues seem to be mediated by diverse modes of action (extensively reviewed in Liongue and Ward 2025). CRLF3 is typically present with single orthologs in placozoa and all eumetazoan taxa from cnidarians to mammals, in contrast to most other cytokine type I receptors that are exclusively present in vertebrates (Wyder et al. 2007; Liongue, Sertori, and Ward 2016; Hahn et al. 2019). CRLF3 belongs to group 1 of cytokine type I receptors, which also includes erythropoietin receptor (EpoR), thrombopoietin receptor (c‐Mpl, TpoR), prolactin receptor (PrlR) and growth hormone receptor (GHR). CRLF3 is located on human chromosome 17, is expressed in most human tissues including the nervous system, and has been associated with differentiation, maturation, cell survival (Wegscheid et al. 2021; Taznin et al. 2022), neuronal dendrite growth (Wilson et al. 2023), immune functions (Yan et al. 2023) and various diseases including amyotrophic lateral sclerosis (ALS) (Cirulli et al. 2015) and autism spectrum disorders (Wegscheid et al. 2021). Highly conserved *Crlf3* orthologs were identified in mammals and various insects such as the beetle *Tribolium castaneum* , the cricket *Gryllus bimaculatus*, and the locust *Locusta migratoria*, but not in the fly *Drosophila melanogaster* (Wyder et al. 2007; Hahn et al. 2019). Previous studies with locust and beetle neurons, as well as human iPSC‐derived neurons, identified CRLF3 as a receptor that promotes cell survival under apoptogenic conditions. The cytokine erythropoietin (Epo) stimulates erythropoiesis and cell protection in vertebrates via its specific receptor EpoR. With respect to the mammalian nervous system, alternative Epo receptors, besides EpoR, mediate neuroprotection (Ostrowski and Heinrich 2018). Although insects lack Epo, both insect and human CRLF3 are activated by human Epo (Hahn et al. 2017; Hahn et al. 2019; Knorr et al. 2023). Importantly, both are also activated by the neuroprotective but non‐erythropoietic human Epo splice variant EV‐3, which is unable to activate the “classic” vertebrate Epo‐Receptor (Miljus et al. 2017; Knorr et al. 2023; Bonnas et al. 2017). Hence, human CRLF3 represents a neuroprotective receptor for Epo and EV‐3, which might be responsible for various previously reported beneficial functions of these cytokines, rendering it a promising target to counteract degenerative cell loss in the nervous system and other tissues.

We hypothesize that specific hematopoietic cytokine receptors evolved from CRLF3 in early vertebrates, particularly in tissues like the nervous system. In order to retain general survival functions, newly evolved cytokines preserved their capacity to activate CRLF3 while additionally binding novel, differentially expressed, and more specific cytokine receptors. Centerpiece to this hypothesis is Epo, which selectively activates EpoR_2_ (homodimeric Epo receptor) on erythrocyte precursors and other cell types. While Epo also mediates cell protection via CRLF3, it cannot activate other vertebrate‐specific cytokine type I receptors (Epo does not activate TpoR and vice versa (Broudy et al. 1997)). To substantiate the hypothesis that CRLF3 is the ancestral receptor of vertebrate type I cytokine receptors, we tested small peptides previously known to elicit cell protection via EpoR_2_‐dependent and EpoR_2_‐independent mechanisms for their ability to activate CRLF3. Additionally, we tested the endogenous ligands of all vertebrate group 1 cytokine type I receptors, namely Tpo, Prl, and GH (in addition to previously studied Epo and EV‐3), for their ability to mediate cell protection via activation of CRLF3. A summary of previously reported functions of these ligands in hematopoiesis, neuroprotection, and protection of other cell types is provided in the supplement (Table S1). Sequences of Epo and EV‐3 and the relation of Epo‐mimetic peptides to these are displayed in Figure S1.

Insect and mammalian CRLF3 are highly conserved, and both have been shown to initiate anti‐apoptotic mechanisms upon stimulation with human Epo and human EV‐3 (Miljus et al. 2017; Knorr et al. 2023). To exclude interfering effects mediated via other cytokine receptors, experiments within this study were conducted with brain neurons of *Locusta migratoria* which endogenously express CRLF3 but none of the vertebrate‐specific hematopoietic cytokine receptors. We demonstrate that short‐peptide Epo mimetics which can activate mammalian EpoR (Epo, EMP1) and those that cannot activate mammalian EpoR (EV‐3, HBSP, P16) all initiate neuroprotection of locust neurons, demonstrating a broader ligand spectrum of CRLF3 than EpoR. Moreover, Tpo but not GH and prolactin protect locust neurons via activation of CRLF3, suggesting that mammalian CRLF3 may also be activated by Epo and Tpo, whereas EpoR and TpoR are selective for their designated ligand. Overall, these results indicate a generalist ligand spectrum of CRLF3, in line with the hypothesis that CRLF3 might be the common ancestor of the more specific cytokine type I receptors.

## 2 Materials and Methods

### 2.1 Cytokines, Epo‐Mimetic Peptides and Inactive Controls

Cytokines (Epo, Tpo, Prl and GH) were purchased from commercial suppliers (Table 1). EV‐3, a splice variant of Epo that lacks amino acids encoded by the third exon (Bonnas et al. 2017), was generated and provided by IBA GmbH (Göttingen, Germany). Epo‐mimetic peptides were synthesized in the Neuroproteomics Group of the Max Planck Institute for Multidisciplinary Sciences by standard solid‐phase peptide synthesis using fluorenylmethoxycarbonyl (Fmoc) chemistry. For Epo A‐helix‐derived P16 (Bonnas 2009) and Epo B‐helix‐derived Helix B surface peptide (HBSP, Brines et al. 2008), scrambled peptides (i.e., same amino acid composition in different order) were synthesized as biologically inactive negative controls. L‐pyroglutamic acid pentachlorophenyl ester (Novabiochem) with ethyl cyano (hydroxyimino)acetate (Oxyma Pure, Novabiochem) activation in dimethylformamide (DMF) was used to introduce the N‐terminal pyro‐glutamic acid residue (pE) into HBSP. A derivative of EMP1, a peptide not related to the primary sequence of Epo (Livnah et al. 1996; Wrighton et al. 1996), was synthesized as above, and the purified peptide was cyclized via disulfide bond formation with the mild oxidant N‐chlorosuccinimide in aqueous solution (Postma and Albericio 2013). The corresponding linear peptide, obtained by reduction of the disulfide bonds and subsequent cysteine alkylation with iodoacetamide, served as a negative control, as the intact disulfide bond is known to be required for the erythropoietic activity of EMP1. All peptides were cleaved from the resin under standard conditions, purified by preparative reversed‐phase high‐performance liquid chromatography (HPLC), and their purity and identity confirmed by analytical HPLC and mass spectrometry.

**TABLE 1:** Cytokines, Epo‐mimetic peptides, and control peptides.

Peptide	Trade name/sequence	Origin
Erythropoietin	NeoRecormon Natural, glycosylated, 165 amino acids	Roche, Welwyn Garden City, UK Cat. No. PZN 08778058
EV‐3	Splice variant that lacks 87 amino acids encoded by the third exon of the human Epo gene	IBA GmbH, Göttingen, Germany
Thrombopoietin (Tpo)	Natural, glycosylated, 332 amino acids	Miltenyi Biotec, Bergisch Gladbach, Germany Cat. No. 130‐095‐745
Prolactin (Prl)	Natural, 198 amino acids	Sigma Aldrich, Munich, Germany Cat. No. L4021
Growth hormone (GH)	Natural, glycosylated, 191 amino acids	PeproTech, Hamburg, Germany Cat. No. 10040100UG
HBSP	pEEQLERALNSS	Neuroproteomics Group of the Max Planck Institute for Multidisciplinary Sciences, Göttingen, Germany
HBSP*	pESAQELRESLN	
P16	APPRLICDSRVLERYL	
P16*	LYPASPIVLDLRRRCE	
EMP1	GGTYSCHFGPLTWVCRPQGG (cyclic)	
EMP1*	GGTYSCHFGPLTWVCRPQGG (linear)	

*Note:* Control peptides are indicated by “*”. HBSP* and P16* contain the same amino acids as HBST and P16, respectively, but in a different order. EMP1* is a linearized peptide with the same primary sequence as EMP1. All peptides were synthesized with a carboxy‐terminal amide group.

### 2.2 Primary Cultures of Locust Brain Cells

Primary cultures were established from both sexes of fourth‐instar nymphs of *Locusta migratoria* (proinsects GMBH, Minden, Germany, Cat. No. PI0110). Until usage, the animals were housed in groups at 24°C temperature, 55% humidity, 12/12 h light/dark cycle, and fed with wheat seedings and lettuce leaves *ad libitum*. Locust brains without optic lobes were dissected and transferred into sterile growth medium consisting of Leibovitz's L‐15 medium (Gibco, Life Technologies, Karlsruhe, Germany, Cat. No. 11415049) supplemented with 1% penicillin/streptomycin (Gibco, Paisley, Scotland, UK, Cat. No. 15140148) and 1% amphotericin B (Gibco, Paisley, Scotland, UK, Cat. No. 15290026). After rinsing with fresh growth medium, extracted brains were treated with growth medium containing 1 mg/mL collagenase and dispase (Sigma‐Aldrich, Cat. No. 10269638001) for 30 min at 27°C to digest extracellular matrix proteins. Following several washing steps with Hanks Balanced Salt Solution (Gibco, Paisley, Scotland, UK, Cat. No. 14170088), brain tissue was triturated through the tip of a 100 μL pipette to dissociate the brains and create a homogenous cell suspension. Cells from all brains used in the same experiment (typically from 2 brains per established culture) were mixed in this step. The resulting cell suspension was centrifuged at 300×*g* for several minutes (QuickSpin 700, Süd‐Laborbedarf, Gauting, Germany) and the cell pellet was resuspended in growth medium. The dissociated cells were evenly dispersed onto concanavalin‐coated round (∅ 10 mm) coverslips (Concanavalin A, Sigma‐Aldrich, Cat. No. C5275), which were placed in sterile plastic culture dishes (∅ 35 mm, Corning Inc., Sigma‐Aldrich). After allowing attachment of the cells to the coated coverslips for 2 h, the culture dishes were filled with growth medium that contained 5% fetal calf serum (FCSG, GE Healthcare, Cat. No. A15‐151). The cell cultures were maintained at 27°C in a humidified normal atmosphere (Incubator BD115, Binder, Tuttlingen, Germany). Medium was changed every other day. Cell cultures were maintained for 5 days before experiments started. Cells were fixed and labeled after 7 days in vitro. At this time, the primary cultures contained > 95% neurons, as determined by previous studies with neuron‐specific anti‐HRP immunolabeling (Gocht et al. 2009; Ostrowski et al. 2011) and regular visual inspection by phase contrast light microscopy in the present studies.

### 2.3 Knockdown of *Crlf3* Expression

In order to reduce levels of CRLF3 protein, locust primary brain neuron cultures were subjected to “soaking RNA interference” with *crlf3*‐specific double‐stranded RNA (dsRNA) as previously described (Hahn et al. 2019; Knorr et al. 2021). This method was initially validated by using two dsRNA molecules that targeted different non‐overlapping fragments of *L. migratoria Crlf3* (Hahn et al. 2019). While dsRNA‐mediated knockdown of *crfl3* expression had no direct impact on neuron survival in normoxia, both dsRNAs targeting different fragments of the *CRLF3* sequence abolished Epo‐mediated neuroprotection under hypoxic conditions. In this study, dsRNA targeting fragment 1 of the initial study was used. Media of respective neuron cultures were supplemented with 10 ng/mL dsRNA immediately after culture establishment. Fresh dsRNA was applied with each medium change and dsRNA exposure was maintained throughout the entire experimental period.

### 2.4 Neuronal Survival in Hypoxia

Cultures of locust brain neurons were exposed to serum‐free medium with or without added compounds for 12 h in normoxia. Since serum contains various factors that promote cell survival, it was omitted to prevent masking of protective effects by the studied peptides. For each experiment, two control cultures (normoxia control and hypoxia control) were incubated with 2 mL serum‐free medium only. Experimental cultures were supplemented with cytokines and Epo‐mimetic peptides at different concentrations as described in the results section. After 12 h, cultures (except for normoxia control) were placed in an airproof chamber (MIC‐101, Billups‐Rothenberg Inc., Del Mar, CA, USA) equipped with an oxygen analyzer (Greisinger GOX 100, Conrad Electronics, Hirschau, Germany). Oxygen (O_2_) was replaced by floating the chamber with nitrogen (N_2_). Hypoxia (O_2_ level ≤ 0.5%) was applied to the cell cultures for 36 h at room temperature. Normoxic control cultures were placed next to the hypoxia chamber to assure identical temperature.

### 2.5 Assessment of Neuronal Survival

**FIGURE 1:**
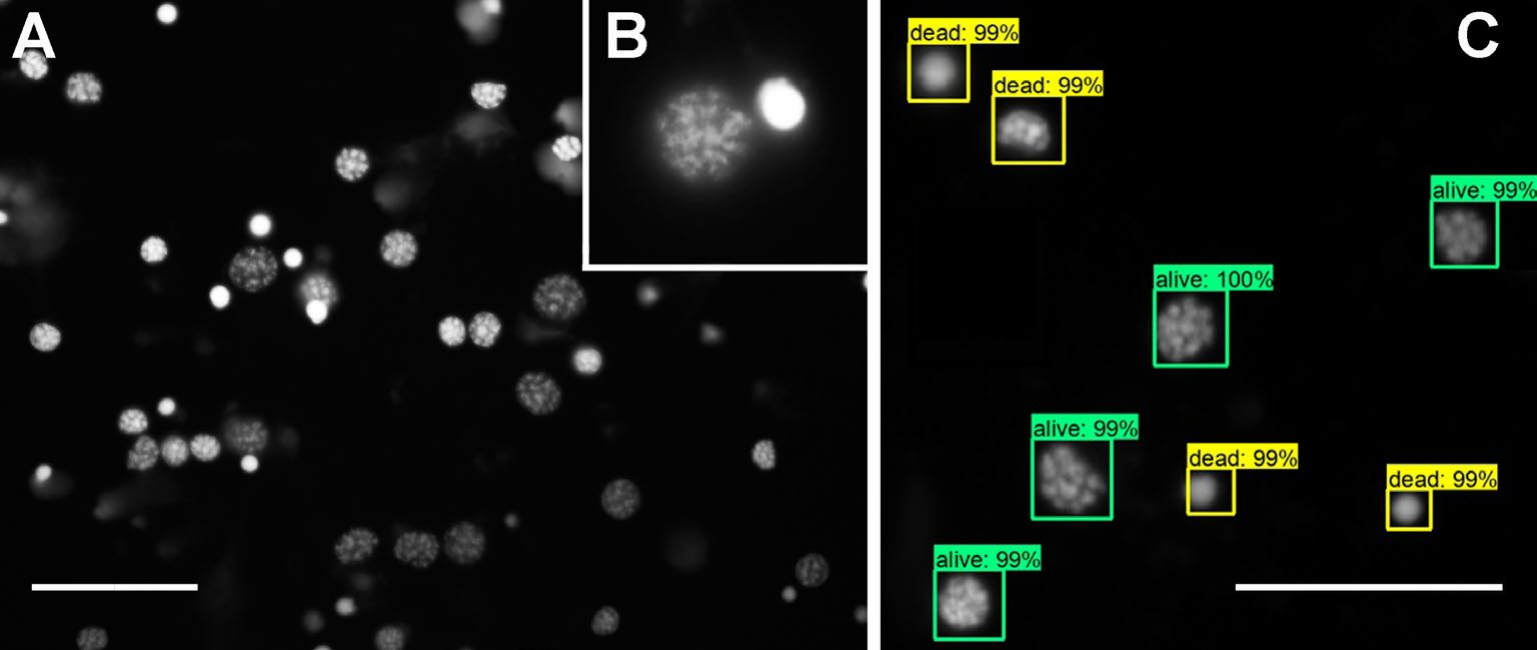
DAPI nuclear staining for live/dead evaluation. (A) Nuclei of intact cells have larger diameters and display patchy fluorescent patterns that reflect the chromatin structure of the DNA. Nuclei of dead or dying cells are condensed and display intense uniform fluorescence. (B) Enlarged nuclei of one intact (left) and one dead (right) cell. (C) Nuclei of intact (green frame) and dead/dying (yellow frame) neurons as scored by the trained convolutional neural network. Percentages indicate the certainty (maximum = 100%) of the classification as “intact” or “dead/dying” cell. Scale bars: 100 μm.

After hypoxia exposure, cell cultures were maintained for 12 h in normal atmosphere before fixation with paraformaldehyde (4% in PBS, 30 min at room temperature). After several washes with PBS (pH 6.9) and PBST (with 0.1% Triton‐X‐100), the cells were incubated with 4′,6‐diamidino‐2‐phenylindole, dihydrochloride (DAPI, Sigma Aldrich, Cat. No. D9542) dissolved in PBST at a final concentration of 100 μg/mL for 30 min in the dark. Finally, excess dye was removed by several washes with PBS, and cell‐containing coverslips were mounted on microscopic slides in 1,4‐diazobicyclo[2.2.2]octane (DABCO, Roth, Karlsruhe, Germany, Cat. No. 0718.1). Analysis of DAPI fluorescence was performed using an epifluorescence microscope (Zeiss Axioskop; 40× objective) equipped with a camera (Spot CCD camera, Invisitron, Puchheim, Germany or Orca Spark, Hamamatsu Photonics, Japan). From each culture, two continuous rows of non‐overlapping photographs (usually around 60–80 per culture) to the right and the left of the center extending over the entire coverslip were taken. Analysis of cell survival (at the time of fixation) was based on DAPI labeled chromatin structure (Figure 1). As previously reported for locusts (Gocht et al. 2009, Ostrowski et al. 2011; Knorr et al. 2020), other insect species (Chao and Nagoshi 1999; Cavaliere et al. 1998; Hahn et al. 2017) and mammalian CNS‐derived cell cultures (Hu et al. 2002; Daniel and DeCoster 2004), DAPI staining distinguishes intact from apoptotic and dead cells.

Series of photographs depicting DAPI‐stained locust brain neurons were used to quantify cellular survival following different experimental treatments. In each culture, DAPI‐labeled nuclei of intact neurons and of dead/dying neurons (Figure 1A,B) were individually counted and the portion of intact neurons (number of intact cells divided by total number of cells) determined. In experiments with Epo‐mimetic peptides, cell counting was performed by a “blinded” observer who had no knowledge about the identity of the culture. In experiments with hematopoietic cytokines, DAPI‐labeled nuclei were counted and identified as “intact” or “dead/dying” by a trained convolutional neural network as described in a previous in vitro study with locust neurons (Knorr et al. 2022, Figure 1C). In brief, DAPI‐stained nuclei were classified as alive or dead using a convolutional neural network with transfer learning from a ResNet‐50 or backbone (He et al. 2016). The classifier was retrained on 1820 annotated images (1456 for training, 364 for testing) containing 25 422 nuclei (20 260 for training, 5182 for testing), all labeled by six independent human experts. Based on DAPI fluorescence, the network integrates criteria such as nuclear morphology and staining intensity, achieving > 98% accuracy on the test set. Manual thresholds (e.g., nucleus size or intensity cutoffs) were not used; instead, the model synthesizes expert‐labeled features for classification.

For each experiment (differently treated cultures contained pooled neurons derived from the same locust brains, see above), percentages of intact cells from all treatment groups were normalized to the normoxic control culture of the same experiment (set to 100%). In this and previous studies, the impact of hypoxia on neuron survival varied considerably. Though this has not been explicitly studied, pre‐exposure of the locusts to physiological stress (e.g., temperature stress, mild hypoxia, enclosure in dark packages during shipment per mail) might affect the susceptibility of the brain neurons to experimentally applied hypoxic stress. Such preconditioning has been demonstrated in mice, whose neurons increased hypoxia‐tolerance following periods of mild hypoxia and other stressors (“cross modality tolerance”). Pooling neurons from the same locust brains in all cultures directly compared with each other, and normalizing neuron survival to the normoxic control culture derived from the same neuron pool largely compensate “experience‐related” differences in hypoxia tolerance.

### 2.6 Statistical Analysis

Data analysis and statistics were performed with R Studio (version 1.2.1335) (R_Core_Team 2019; RStudio_Team 2018). Boxplots depict the median; the upper and lower quartile; whiskers represent 1.5 times the interquartile range; and outliers. Black and colored circles represent the data of individual experiments. Since data were not normally distributed in all treatment groups of all experiments, we generally used non‐parametric tests for statistical evaluation of all experiments. Statistics were calculated using the pairwise permutation test without outlier exclusion included within the packages “coin” and “rcompanion” (Hothorn et al. 2008; Hothorn et al. 2006; Mangiafico 2019). The false discovery rate was controlled using the Benjamini‐Hochberg procedure (Benjamini and Hochberg 1995).

## 3 Results

### 3.1 Small Epo‐Mimetic Peptides Protect Locust Neurons From Hypoxia‐Induced Cell Death

**FIGURE 2:**
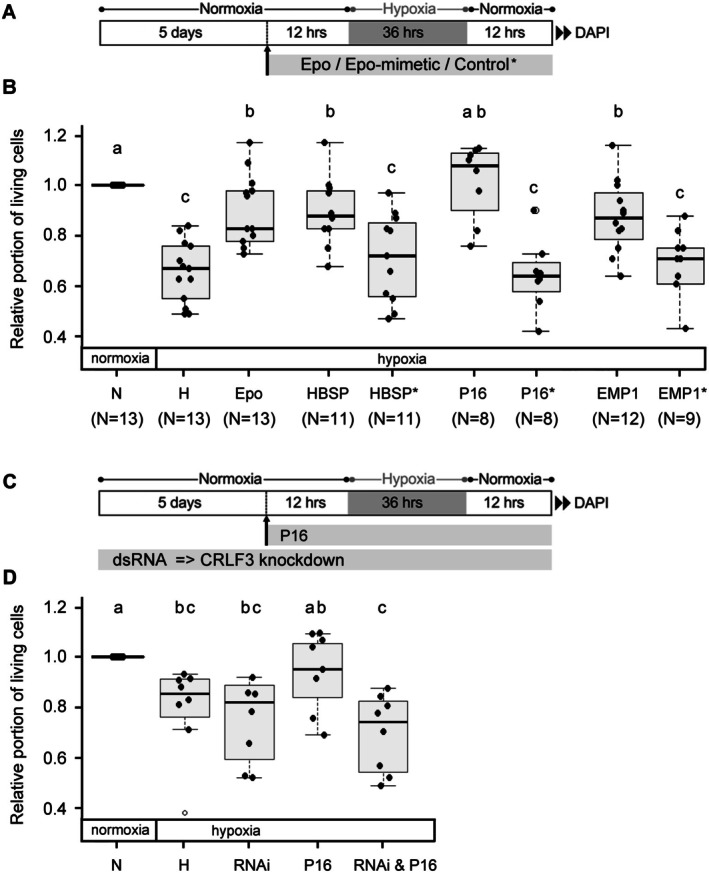
Impact of Epo and Epo‐mimetics on neuronal survival in hypoxia. (A) Experimental procedure. (B) Hypoxia (H) reduces in vitro cellular survival compared to normoxic control (N). Hypoxia‐induced cell death is significantly reduced by Epo (33.3 ng/mL, *p* = 0.004), HBSP (0.11 ng/mL, *p* = 0.004), P16 (6.65 ng/mL, *p* = 0.002) and EMP1 (6.65 ng/mL, *p* = 0.006). Only P16‐treated cultures reached the survival level of control cells. Cell survival in cultures supplemented with HBSP, P16, and EMP1 was higher compared to cultures incubated with the same concentration of “inactive” control peptides HBSP*, P16*, and EMP1*. Numbers in brackets represent repetitions of independent experiments with the respective treatment group. 139 988 cells were evaluated in total. (C) Experimental procedure. (D) Hypoxia‐induced cell death is reduced (though not significantly (*p* = 0.180) due to unusually low survival in two experiments) by P16 (6.65 ng/mL). The protective effect of P16 is significantly reduced after dsRNAi‐mediated knockdown of CRLF3 expression (hypoxia & P16 vs. hypoxia & P16 & RNAi, *p* = 0.030). Exposure to dsRNA had no negative impact on hypoxia‐exposed neurons (*p* = 0.628). *N* = 8 independent cell culture preparations. 114 838 cells were evaluated in total. Statistics: Permutation test with Benjamini‐Hochberg correction. Treatment groups that do not share a letter (a, b, c) are significantly different with at least *p* < 0.05.

As previously reported, exposure to hypoxia for 36 h (Figure 2A) initiates apoptosis leading to decreased survival of cultured locust brain cells (Figure 2B). The presence of Epo in culture medium partially reduced hypoxia‐induced cell death, leading to significantly more (*p* = 0.004) surviving cells compared to the hypoxia‐only condition. Suitable concentrations of small Epo‐mimetic peptides (HBSP, P16, EMP1) were estimated from previous studies on mammalian cells. Similar to Epo, all three Epo‐mimetic peptides tested in this study significantly increased survival in hypoxia‐exposed cell cultures (HBSP: *p* = 0.004, EMP1: *p* = 0.006) with P16 showing the strongest neuroprotective effect (*p* = 0.002), fully compensating hypoxia‐induced cell loss. In contrast, none of the control peptides containing the same amino acids in a scrambled sequence (HBSP*, P16*) or as a linearized version of the cyclic active peptide (EMP1*) had a beneficial effect on cell survival in hypoxia‐exposed cultures. These results indicate that the three tested small peptides protect locust brain neurons from hypoxia‐induced cell death. In an additional series of experiments, we tested whether the most potent peptide P16 mediated its protective effects via CRLF3. Locust brain neurons were incubated with dsRNA throughout the entire culture period to inhibit the production of CRLF3 and reduce its levels in the cultured cells (Figure 2C). As seen in the first experiment (Figure 2B), P16 increased the survival of hypoxia‐exposed neurons to reach the level of normoxic controls (Figure 2D). In contrast, P16‐mediated protection was absent in cultures subjected to dsRNA treatment, indicating that CRLF3 is required for the beneficial effect of P16. Reduction of CRLF3 by dsRNA incubation had no negative impact on the survival of hypoxia‐exposed neurons.

### 3.2 Human Epo and Tpo Protect Locust Neurons From Hypoxia‐Induced Cell Death

**FIGURE 3:**
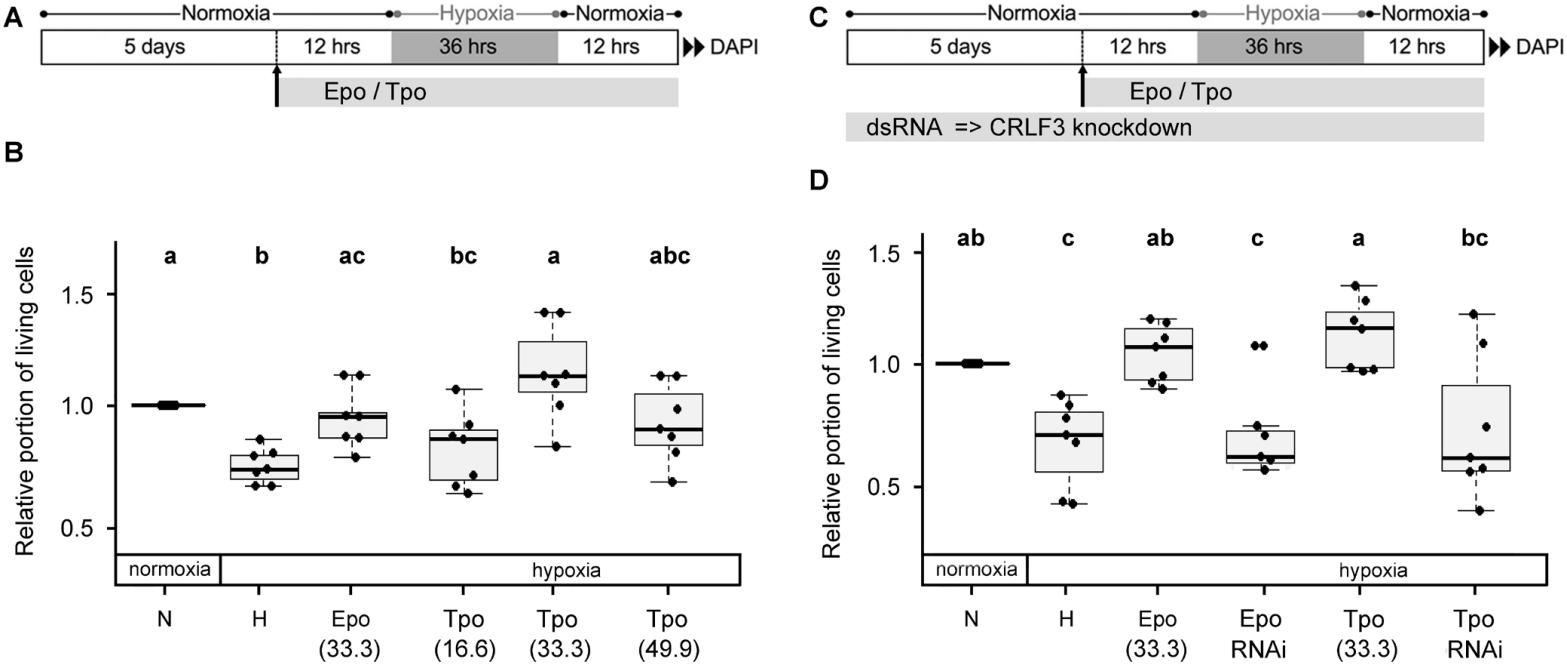
Impact of Epo and Tpo on neuronal survival in hypoxia. (A) Experimental procedure for experiment displayed in B. (B) Hypoxia (H) reduces in vitro cellular survival compared to normoxic control (N). Hypoxia‐induced cell death is significantly reduced by Epo (33.3 ng/mL) and Tpo (33.3 ng/mL). Both 16.6 ng/mL and 49.9 ng/mL Tpo slightly increase neuronal survival in hypoxia. Numbers in brackets represent the concentrations of Epo or Tpo in ng/mL. *N* = 7 independent cell culture preparations. 44 586 cells were evaluated. (C) Experimental procedure for experiment displayed in D. To inhibit CRLF3 production, culture medium was supplemented with 10 ng/μL dsRNA throughout the entire culture period. (D) Hypoxia (H) reduces in vitro cellular survival compared to normoxic control (N). Hypoxia‐induced cell death is significantly reduced by Epo (33.3 ng/mL) and Tpo (33.3 ng/mL). Protective effects of both cytokines are absent after RNAi‐mediated knockdown of CRLF3 expression. Numbers in brackets represent the concentrations of Epo or Tpo in ng/mL. *N* = 7 independent cell culture preparations. A total of 30 808 cells were evaluated. Statistics: Permutation test with Benjamini‐Hochberg correction. Treatment groups that do not share a letter (a, b, c) are significantly different with at least *p* < 0.05.

Since both human Epo (erythropoietic via EpoR_2_ and neuroprotective via EpoR_2_ and other alternative receptors) and its splice variant EV‐3 (non‐erythropoietic but neuroprotective without involvement of EpoR) activate insect and human CRLF3, we studied the potential activation of locust CRLF3 by other human type 1 cytokines. As shown in Figure 3, hypoxia significantly reduced neuronal survival (*p* = 0.011) and Epo protected locust brain neurons from hypoxia‐induced apoptosis (*p* = 0.024). Hypoxia‐exposed neurons derived from the same pool of locust brains were also treated with three different concentrations of Tpo. Neuron survival in hypoxic conditions significantly increased by the addition of 33.3 ng/mL Tpo (*p* = 0.0244), while both lower (16.6 ng/mL; *p* = 0.29) and higher (49.9 ng/mL; *p* = 0.066) concentrations showed only trends toward enhanced survival; however, they did not reach statistical significance (Figure 3). The results indicate comparable protective potencies of Tpo and Epo, with both cytokines having a similar optimum‐type dose curve for their anti‐apoptotic effects on locust neurons.

### 3.3 Human Epo and Tpo Mediate Locust Neuroprotection via CRLF3

In order to determine whether Epo and Tpo promote locust neuron survival via activation of CRLF3, expression of the receptor was inhibited by incubation of neuronal cultures with dsRNA for 5 days prior to hypoxia exposure (Figure 3C). As shown in Figure 3D, both Epo (*p* = 0.013) and Tpo (*p* = 0.011) prevented hypoxia‐induced death of locust brain neurons. Protective effects of both human cytokines were significantly reduced in cultures subjected to dsRNA treatment to reduce the level of CRLF3 (Epo: *p* = 0.013 and Tpo: *p* = 0.036). This indicates that both Epo and Tpo mediate their protective effects in locust brain neurons via activation of CLRF3.

### 3.4 Human Growth Hormone (GH) Does Not Alter the Survival of Hypoxia‐Exposed Locust Brain Neurons

**FIGURE 4:**
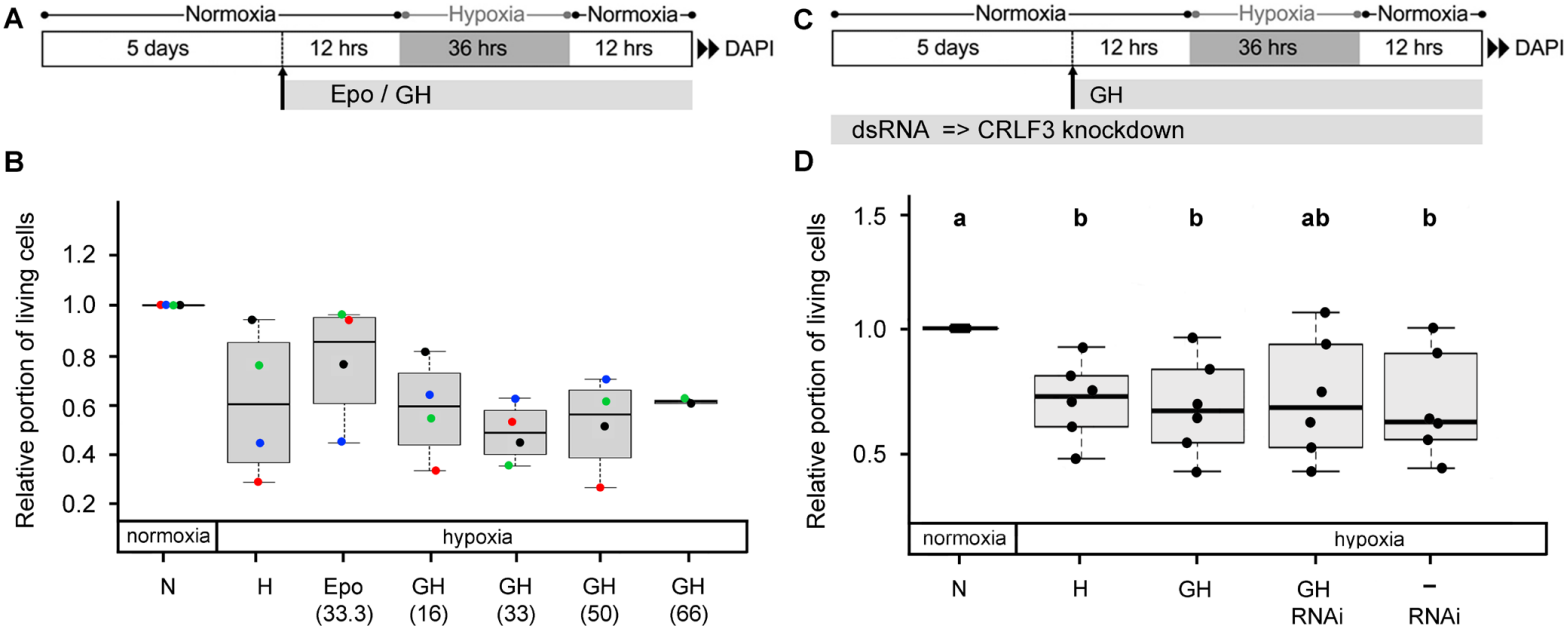
Impact of GH on neuronal survival in hypoxia. (A) Experimental procedure for the experiment displayed in B. (B) Hypoxia (H) reduces in vitro cellular survival compared to normoxic control (N). Hypoxia‐induced cell death is reduced by Epo (33.3 ng/mL) in three out of four experiments. Medium neuron survival of GH‐treated and hypoxia‐exposed cultures is lower or on the same level as survival in untreated hypoxic control cultures. Numbers in brackets represent the concentrations of Epo or GH in ng/mL. Colored dots indicate values from a particular experiment. *N* = 4 independent cell culture experiments except *N* = 2 for GH in the highest concentration. 53 005 cells were evaluated. (C) Experimental procedure for the experiment displayed in D. To inhibit CRLF3 production, culture medium was supplemented with 10 ng/μl dsRNA throughout the entire culture period. (D) Hypoxia (H) reduces in vitro cellular survival compared to normoxic control (N) (*p* = 0.026). Hypoxia‐induced cell death is unaffected by GH (20 ng/mL). RNAi‐mediated knockdown of CRLF3 expression had no impact on cell survival in cultures subjected to hypoxia, with or without GH treatment. Numbers in brackets represent the concentrations of Epo or Tpo in ng/mL. *N* = 6 independent cell culture preparations. 91 377 cells were evaluated. Statistics: Permutation test with Benjamini‐Hochberg correction. Treatment groups that do not share a letter (a, b) are significantly different with at least *p* < 0.05.

Experiments with different concentrations of human (GH ranging from 16 to 66 ng/mL) revealed no particular protective effect on hypoxia‐exposed neurons (Figure 4A,B). We selected a concentration of 20 ng/mL, containing a similar number of ligand molecules per volume as 33 ng/mL Epo (the most neuroprotective concentration determined in previous studies), for studying the potential anti‐apoptotic effect of GH via CRLF3 (Figure 4C,D). Exposure to hypoxia significantly reduced (*p* = 0.026) neuronal survival (Figure 4D). Hypoxia‐induced cell death was not prevented by GH and/or RNAi‐mediated knockdown of CRLF3 expression, indicating that GH cannot activate locust CRLF3.

#### 3.4.1 Human Prl Does Not Alter the Survival of Hypoxia‐Exposed Locust Brain Neurons

**FIGURE 5:**
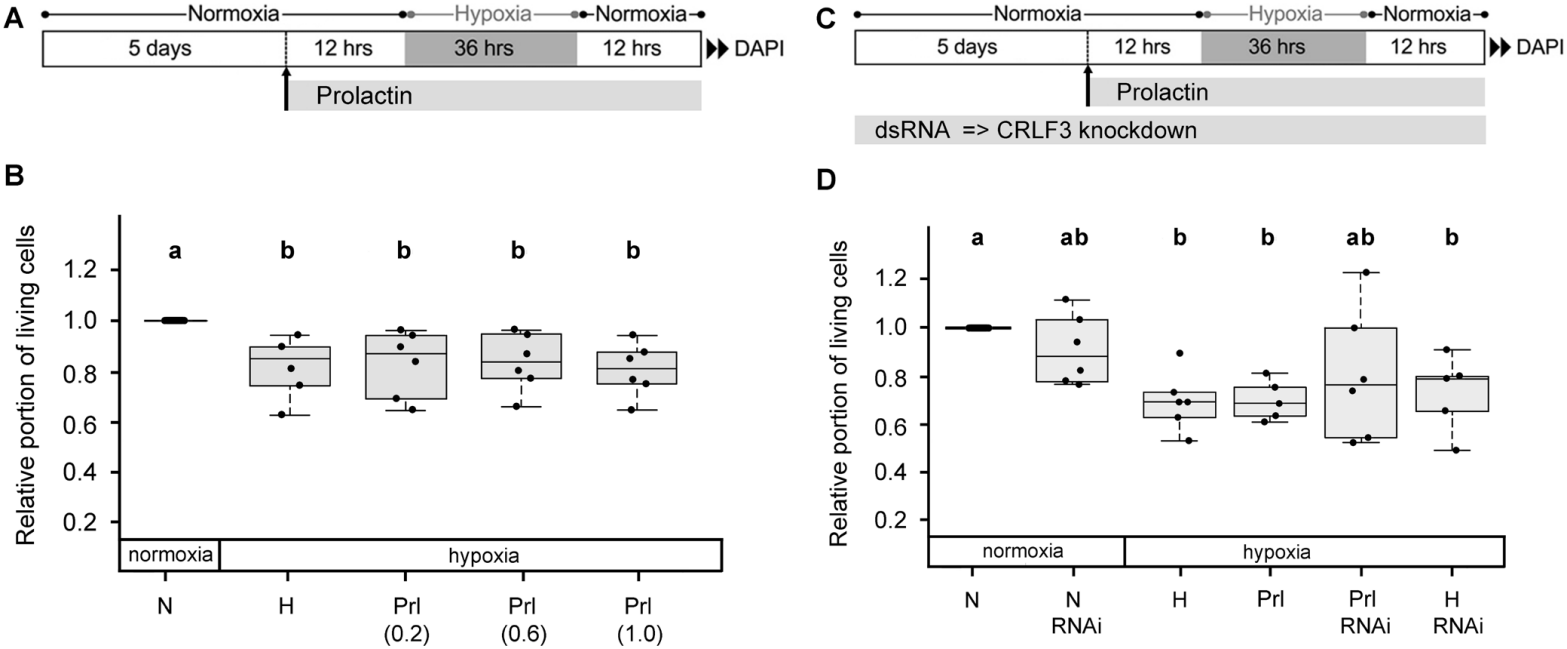
Impact of Prl on neuronal survival in hypoxia. (A) Experimental procedure for experiment displayed in B. (B) Hypoxia (H) reduces in vitro cellular survival compared to normoxic control (N). Hypoxia‐induced cell death is unaffected by three concentrations of Prl (0.2 ng/mL; 0.6 ng/mL and 1.0 ng/mL). Medium neuron survival in Prl‐treated and hypoxia‐exposed cultures is similar to survival in untreated hypoxic control cultures. Numbers in brackets represent the concentrations of Prl in ng/mL. *N* = 6 individual cell culture preparations except *N* = 5 for hypoxia control. 83 862 cells were evaluated. (C) Experimental procedure for experiment displayed in D. To inhibit CRLF3 production, culture medium was supplemented with 10 ng/μL dsRNA throughout the entire culture period. (D) Hypoxia (H) reduces in vitro cellular survival compared to normoxic control (N) (*p* = 0.023). Hypoxia‐induced cell death is unaffected by Prl (0.2 ng/mL). RNAi‐mediated knockdown of CRLF3 expression had no impact on cell survival in normoxic control cultures and in cultures subjected to hypoxia. Neuron survival in hypoxia‐exposed cultures that received Prl and dsRNA was neither significantly different from normoxic nor from hypoxic controls due to large variation of relative survival in individual experiments. *N* = 6 independent cell culture preparations. 118 721 cells were evaluated. Statistics: Permutation test with Benjamini‐Hochberg correction. Treatment groups that do not share a letter (a, b) are significantly different with at least *p* < 0.05.

A series of experiments with three different concentrations of Prl (0.2, 0.6, and 1.0 ng/mL) revealed no significant protective effect on hypoxia‐exposed locust brain neurons (Figure 5A,B). Since no studies with Prl on insect cells were found in the literature, Prl concentrations in these experiments were chosen according to the range of EC_50_ concentrations (0.03–0.3 ng/mL) from previous in vivo studies on vertebrates recommended by the manufacturer. Based on the results shown in Figure 5B we selected the concentration with the highest median value of relative neuronal survival (0.2 ng/mL) for further experiments that also included dsRNA‐mediated knockdown of CRLF3 expression (Figure 5C). Consistent with experiments described above, exposure of neuronal cultures to hypoxia significantly reduced the portion of intact cells compared to normoxic control cultures (*p* = 0.023) (Figure 5D). However, neuron survival in hypoxia was not improved by treatment with Prl that was initiated 12 h before the start of the hypoxic period. Incubation of normoxic and hypoxic cultures with dsRNA increased the variability of relative cellular survival without significantly altering the proportion of intact cells. Hence, no neuroprotective effect of Prl on locust brain neurons could be detected.

## 4 Discussion

Epo has a widely acknowledged neuroprotective potential for clinical application in degenerative and neuropsychiatric diseases (Rey et al. 2019; Miskowiak et al. 2016; Fond et al. 2012). However, since its specific receptor EpoR is also expressed in the hematopoietic system and in certain tumors, potential benefits of Epo treatment in the nervous system are invariably accompanied by serious side effects (Ehrenreich et al. 2009; Cao 2013). In order to exploit the neuroprotective functions of Epo, alternative Epo receptors, co‐expressed with classical EpoR in the nervous systems but absent from hematopoietic cells, need to be identified. To the present, alternative mammalian Epo receptors involved in neuroprotection include the β‐cR/EpoR heteroreceptor, Ephrin B4 receptor, and CRLF3 (Ostrowski and Heinrich 2018). Besides Epo itself and synthetic molecules (carbamylated Epo, HBSP), no specific endogenous ligand has been identified for the heterodimeric β‐cR/EpoR (Leist et al. 2004; Brines et al. 2008; Brines 2010). Human CRLF3 was demonstrated to mediate neuroprotection of iPSC‐derived neurons upon stimulation with both Epo and its non‐erythropoietic splice variant EV‐3 (Knorr et al. 2023). Thus, signaling via EV‐3 (instead of Epo) can specifically target cell protective mechanisms without stimulating erythropoiesis. CRLF3 is widely expressed in mammalian nervous systems and other tissues and may account for a portion of previously described protective functions of Epo (Rey et al. 2019; Vittori et al. 2021). The present study extended the range of already known CRLF3 ligands by three previously studied small‐peptide Epo‐mimetics and thrombopoietin.

The present study used brain neurons of the insect *Locusta migratoria* that express CRLF3 but no other (vertebrate‐specific) class I cytokine receptors; therefore, allowing a clear assessment of CRLF3‐mediated neuroprotective mechanisms. Insect and mammalian CRLF3 contain highly similar structural domains and share 29% overall similarity in their amino acid sequences (Hahn et al. 2019). While the stoichiometry of the functional receptor is still unresolved, CRLF3 receptors of both animal taxa activate intracellular Janus kinase and induce STAT transduction pathways (Miljus et al. 2014; Yang et al. 2009). Both insect and human CRLF3 mediate neuroprotection upon stimulation with human Epo and human EV‐3 (Miljus et al. 2017; Knorr et al. 2023). Most importantly, insects express CRLF3 but none of the other members of group 1 cytokine type I receptors; hence, human Epo and EV‐3 mediate their protective functions on locust neurons exclusively via CRLF3. This was also confirmed for Tpo and P16 by reduction of their neuroprotective effects following RNAi‐mediated knockdown of CRLF3 expression.

Previous studies on insect CRLF3 revealed optimum‐type dose dependencies for its stimulation with Epo and EV3. Neuroprotective effects initially increase with ligand concentrations, then decrease after reaching the optimal concentration and even reverse to toxic impact at very high concentrations (Heinrich et al. 2017; Knorr et al. 2022). Similar dose–response characteristics have been described for various vertebrate cytokine receptors, including receptors for Epo (Siren et al. 2001; Chong et al. 2003; Weishaupt et al. 2004), prolactin (Yang et al. 2009) and granulocyte‐colony stimulating factor (G‐CSF, Jung et al. 2006; Frank et al. 2009). The mechanisms underlying the switch from protection by moderate concentrations to loss of protective or even toxic effects of high ligand concentrations are currently unknown. Optimal protective ligand concentrations seem to depend on cell type, species, and physiological state of the cytokine‐exposed cells. Contradictory results on pro‐ versus anti‐apoptotic effects of Tpo in the vertebrate nervous system may relate to similar mechanisms (Ehrenreich et al. 2005; Li et al. 2020). In order to detect a potential neuroprotective function mediated via locust CRLF3, we typically tested several different concentrations of a particular ligand. Concentrations of human cytokines (Tpo, GH, Prl) were centered around a concentration (in molecular weight/volume) that provided a number of ligand molecules per volume similar to the numbers of Epo molecules in its most protective concentration. For locust primary brain neurons, the most protective concentration of Epo was determined to be ~33 ng/μl (equivalent to 4 U/mL) in previous studies (Ostrowski et al. 2011). CRLF3‐mediated neuroprotective effects of Tpo seem to have a similar dose dependency as Epo, since 33.3 ng/mL was most effective, while both higher and lower concentrations of Tpo were less protective. Suitable concentrations of small Epo‐mimetic peptides (HBSP, P16, EMP1) were estimated from previous studies on mammalian cells. Since the initially selected concentrations induced protective effects, experimental series with different concentrations were not performed. Hence, although significant protection of hypoxia‐exposed locust neurons was statistically confirmed, maximal neuroprotection may be achieved with a different concentration of the respective peptide.

Several non‐erythropoietic but neuroprotective Epo‐related ligands have been previously generated. Some of them, like carbamylated Epo protein (cEpo) (Leist et al. 2004; Chamorro et al. 2013), target the heteromeric β‐cR/EpoR, which is also activated by Epo but not by EV‐3 (Bonnas et al. 2017). Recently, the human ortholog of the evolutionary conserved CRLF3 has been identified as a neuroprotective receptor for both Epo and EV‐3 (Knorr et al. 2023). Using insect neurons that endogenously express CRLF3 but no other vertebrate cytokine type 1 receptor, we demonstrate in this study that various synthetic short peptide Epo‐mimetics prevent apoptosis. Involvement of CRLF3 was confirmed for the most potent peptide P16, whose protective effect vanished with RNAi‐mediated knockdown of CRLF3 expression. The three peptides used in this study are related to Epo to different degrees: P16 represents a portion of the helix A, HBSP consists of a continuous connection of amino acids exposed from the helix B toward the aqueous phase, and EMP1 has no sequence similarity with Epo. While EMP1 stimulates both erythropoiesis and neuroprotection in mammals, P16 and HBSP do not stimulate erythropoiesis via EpoR_2_. HBSP can activate β‐cR/EpoR, whereas a potential receptor for P16 has not been identified, and the neuroprotective functions previously reported for all three Epo‐mimetic peptides might (at least partially) rely on their activation of mammalian CRLF3 (Table S1). Despite their small size of 11–20 amino acids and their limited similarity to full‐size Epo and EV‐3, all three peptides rescued locust neurons from hypoxia‐induced apoptosis. Involvement of CRLF3 as the receptor being activated by the short‐peptide ligands was concluded by the absence of other type I cytokine receptors and reduction of P16‐mediated neuroprotection following knockdown of CRLF3 production. It can therefore be assumed that HBSP and EMP1 also mediated their neuroprotective effects via locust CRLF3. In similar experiments, neuroprotection mediated by Epo and EV‐3 (Hahn et al. 2017, 2019) and Tpo (this study) was demonstrated to depend on CRLF3 activation. In addition to the natural cytokines Epo, EV3, and Tpo, both erythropoietic and non‐erythropoietic small peptides can activate CRLF3, indicating a less specific, more generalist ligand spectrum compared to EpoR.

Type 1 cytokine receptors include five structural groups, with group 1 containing CRLF3, EpoR, TpoR (= c‐MPL), GHR, and PrlR, which are activated by respective long‐chain class 1 cytokines that share a bundle of four helices as a common structural element. While it has been proposed, but not yet experimentally confirmed, that CRLF3 forms homodimers for active signaling (Wilson et al. 2023), functional homodimers have been reported for all other receptors of this group. In addition, EpoR also associates as heteromers with the β‐cR subunit to mediate cell protection in some non‐hematopoietic tissues (Brines and Cerami 2012). Most cytokine receptors of this group are expressed in mammalian brains in addition to their functions in the hematopoietic system. Regarding the latter, EpoR_2_ promotes the differentiation of erythrocytes, TpoR supports megakaryocyte maturation and hematopoietic stem cell maintenance, and both GH and PRL, though having minor impacts on basal hematopoiesis, promote recovery of progenitor cell pools and mature blood cells (Bozhilov et al. 2023; Welniak et al. 2000; Hanley et al. 2005). Similarly, in addition to its presence in the nervous system and other tissues, CRLF3 has been implicated in mouse platelet production (Bennett et al. 2022) and in early definite hematopoiesis of zebrafish (Taznin et al. 2022). CRLF3 is the only known receptor for the non‐erythropoietic Epo splice variant EV‐3, and it can also be activated by full‐length Epo (Knorr et al. 2023). Here we show that Tpo also activates CRLF3 to mediate protection in locust neurons. Tpo and Epo share high similarity of their aminoterminal portions involved in receptor interaction (Lok et al. 1994). Both cytokines activate their designated receptors EpoR and TpoR and cannot cross‐activate the other receptor (Broudy et al. 1997). Nevertheless, both Epo and Tpo (in addition to EV‐3) do activate CRLF3, indicating its less specific ligand profile. In contrast, GH and PRL cannot activate locust CRLF3, though their specific receptors GHR and PrlR also belong to the same group of cytokine type I receptors. Moreover, GH can activate the GH‐receptor and PRL receptor (but not vice versa), demonstrating some similarity in the ligand‐binding domains of both cytokine receptors (Goffin et al. 1996; Brooks 2012). To contextualize our and previous studies of type I cytokines, we turn to the hypothesis that general tissue‐protective functions of hematopoietic cytokines preceded their specific functions in the production and differentiation of blood and immune cells (Ghezzi and Conklin 2013) and the evolutionary ancient *CRLF3* represents the single ancestral cytokine receptor gene that was present at the base of vertebrate evolution (Liongue, Sertori, and Ward 2016; Huising et al. 2006; Ocampo et al. 2009). In support of this, single orthologs of CRLF3 exist in species of all major groups of animals ranging from placozoa to mammalia (Liongue, Sertori, and Ward 2016; Hahn et al. 2017). High conservation of CRLF3, which results from essential and/or pleiotropic functions (He and Zhang 2006; Hu et al. 2017) manifests in similar spectra of activating ligands and anti‐apoptotic mechanisms of mammalian and insect CRLF3 (Knorr et al. 2023; Miljus et al. 2014; this study). While sea squirts (= invertebrate chordates) express CRLF3 but none of the other group 1 cytokine receptors, whole genome duplications at the base of vertebrate evolution (1R and 2R) (Kasahara 2007) and subsequent sequence divergences fostered the emergence of vertebrate‐specific cytokine receptors mediating specialized functions in particular tissues and particular cell types therein (Ocampo Daza and Larhammar 2018). Gene duplications, leading to redundant genes, facilitate the adaptation of new functions associated with altered sequences and related structural modifications of encoded proteins. In a first step, genes encoding intermediate receptors EpoR/TpoR and GH‐R/Prl‐R evolved before jawed vertebrates diverged from agnaths (Ocampo Daza and Larhammar 2018; Boulay et al. 2022). After 2R, separate orthologs of EpoR, TpoR, and CRLF3 emerged, as has been documented in zebrafish (Liongue and Ward 2007). Specific receptors for GH and Prl and associated ligands already evolved in agnaths, since they are present in extant lampreys (Li et al. 2014; Ocampo Daza and Larhammar 2018). Duplicated genes that subsequently diverged and produced altered proteins initially appeared in the same cells and tissues. Hence, new cytokine receptors and their ligands that later assumed specific roles in hematopoietic and immune cells evolved in the presence of the old receptor that mediated general adaptive responses to challenging conditions. In this sense, it appears plausible that the new ligands, in addition to activating their co‐evolved specific receptors, retained their capability to stimulate beneficial responses via the ancestral receptor. This might strengthen the hypothesis that CRLF3 represents the ancestral origin of EpoR and TpoR, whose co‐evolved specific ligands Epo and Tpo still activate CRLF3 to initiate mechanisms that secure cell survival and functionality. Concerning signaling via Epo, the splice variant EV‐3, that presumably evolved after Epo, lost its ability to activate EpoR_2_, thereby separating erythropoietic functions from cell protection in the nervous system and other tissues.

In contrast to Epo/EpoR and Tpo/TpoR cytokine signaling that emerged with tetraploidizations in basal (pre‐)vertebrates, the evolution of GH and Prl and their specific receptors involved several lineage‐specific gene gains and losses (Ocampo Daza and Larhammar 2018) which caused larger variability of GH/Gh‐R and Prl/Prl‐R signaling and corresponding larger deviations from their respective molecular ancestors. This is reflected in multiple isoforms of GH and Prl arising through multiple orthologs of their coding genes, alternative splicing, and posttranslational modifications (Chen et al. 1989; Baumann 1991; Sinha 1995; Papper et al. 2009), and alternative splicing‐generated isoforms of GH‐R and Prl‐R (Bernard et al. 2019). The higher variability of GH/Gh‐R and Prl/Prl‐R signaling arising through this could be the basis as to why neither GH or Prl could initiate neuroprotection within our study.

The present study demonstrates that insect CRLF3 is not only activated by the human hematopoietic factor Epo and its non‐hematopoietic splice variant EV‐3, but also by human Tpo and synthetic small peptide Epo mimetics mediating both hematopoietic and non‐hematopoietic effects in mammalian cells. Furthermore, we add evidence that CRLF3 is the original, ancestral cytokine receptor that gave rise to the plethora of cytokine receptors and their versatile functions in vertebrates. Our study further highlights the importance of studying CRLF3 not only in the context of disease and neuroprotection, but especially for its role in the emergence of cytokine signaling during evolution.

## Author Contributions

**Nina Hahn:** conceptualization, writing – original draft, investigation, supervision, writing – review and editing, methodology, visualization. **Debbra Y. Knorr:** investigation, methodology, writing – original draft, visualization, writing – review and editing, supervision. **Björn Twellsieck:** investigation, writing – review and editing. **Ruoyu Huang:** investigation, writing – review and editing. **Abigail Trebilcock:** investigation, writing – review and editing. **Nicola Schwedhelm‐Domeyer:** investigation, writing – review and editing. **Stephanie Pauls:** investigation, writing – review and editing. **Lars v. Werven:** investigation, writing – review and editing, resources. **Olaf Jahn:** conceptualization, investigation, writing – review and editing, methodology, resources. **Hannelore Ehrenreich:** conceptualization, methodology, writing – review and editing. **Ralf Heinrich:** conceptualization, investigation, funding acquisition, writing – original draft, methodology, visualization, writing – review and editing, supervision.

## Ethics Statement

All experiments were conducted in compliance with the ARRIVE guidelines.

## Consent

Informed consent was achieved for all subjects, and the experiments were approved by the local ethics committee.

## Conflicts of Interest

The authors declare no conflicts of interest.

## Peer Review

The peer review history for this article is available at https://www.webofscience.com/api/gateway/wos/peer‐review/10.1111/jnc.70207.

## Supporting information


**Data S1:** jnc70207‐sup‐0001‐DataS1.pdf.

## Data Availability

The data that support the findings of this study are available from the corresponding author upon reasonable request.
