# Supplementary material for: Multiple Mammalian Cytokines and Erythropoietin‐Mimetic Peptides Protect Insect Neurons via Phylogenetically Conserved Cytokine Receptor‐Like Factor 3 (CRLF3)
Source: J Neurochem. 2025 Sep 3;169(9):e70207. doi: 10.1111/jnc.70207 (PMC12409108; doi:10.1111/jnc.70207)
Supplement: Supplementary file 1 — Data S1: jnc70207‐sup‐0001‐DataS1.pdf. [file JNC-169-0-s001.pdf]

Multiple mammalian cytokines and erythropoietin-mimetic peptides protect insect neurons via phylogenetically conserved cytokine receptor-like factor 3 (CRLF3)

Nina Hahn, Debbra Y Knorr, Björn Twellsieck, Ruoyu Huang, Abigail Trebilcock, Nicola Schwedhelm-Domeyer, Stephanie Pauls, Lars v. Werven, Olaf Jahn, Hannelore Ehrenreich, Ralf Heinrich

Supplements:

Ligand	Description	Functions	Reference
Epo	natural ~34 kDa glycosylated helical peptide of 165 amino acids	erythropoietic<br>neuroprotective<br>protection of other cells	1, 2, 3<br>4, 5, 6, 7<br>8, 9, 10, 15
EV-3	natural splice variant of human Epo that lacks 87 amino acids of the AB loop encoded by exon 3	not erythropoietic<br>neuroprotective	16<br>16, 17, 18
Tpo	natural ~ 95 kDa glycosylated peptide of 332 amino acids	proliferation and differentiation of various hematopoietic cells e.g. thrombocytes<br>neuroprotective<br>protection of other cells	19, 20<br>21, 22, 23<br>24, 25
Prl	natural, 198 amino acids; various variants with molecular weights ranging from 14 to 150 kDa	neuroprotection<br>protection of other cells	26, 27, 28<br>29
GH	natural, 191 amino acids, various glycosylated variants between ~20 and ~24 kDa	neuroprotection<br>protection of other cells	30, 31, 32<br>33
HBSP	11 amino acid peptide related to the helix B surface of Epo	not erythropoietic<br>neuroprotective<br>protection of other cells	34, 35<br>35<br>36, 37, 38, 39
P16	16 amino acid peptide that represents a portion of the helix A of human Epo	not erythropoietic<br>neuroprotective	40<br>40
EMP1	20 amino acid peptide with internal disulfide bridge without sequence relation to Epo	erythropoietic<br>neuroprotective<br>protection of other cells	41, 42<br>43

Table S1: Effects of Epo, EV-3, Tpo, Prl, GH and small peptide Epo-mimetics (HBSP, P16, EMP1) in hemopoiesis, neuroprotection and protection of other cell types.

1 Erslev 1953, 2 Jelkmann 1992, 3 Constantinescu et al. 1999, 4 Siren et al. 2001, 5 Dame et al. 2001, 6 Morishita et al. 1996, 7 Ostrowski et al. 2011, 8 Zou et al. 2016, 9 Calvillo et al. 2003, 10 Szenajch et al. 2010, 11 Leist et al. 2004, 12 Chamorro et al. 2013, 13 Ding et al. 2016, 14 Tögel et al. 2016, 15 Erbayraktar et al. 2009, 16 Bonnas et al. 2017, 17 Miljus et al. 2017, 18 Hahn et al. 2017, 19 Geddis et al. 2002, 20 Hitchcock & Kaushansky 2014, 21 Zhou et al. 2012, 22 Wu et al. 1018, 23 Li et al. 2020, 24 Li et al. 2006, 25 Baker et al. 2015, 26Tejadilla et al. 2010, 27 Molina-Salinas et al. 2023, 28 Vergara-Castaneda et al. 2016, 29 Yang et al. 2023, 30 Baltazar-Lava et al. 2022, 31 Martinez-Moreno et al. 2016, 32 Scheepens et al. 2001, 33 Taghizadeh et al. 2024, 34 Ahmet et al. 2011, 35 Yang et al. 2014, 36 Ahmet et al. 2013, 37 Zhang et al. 2017, 38 Yang et al. 2013, 39 Brines et al. 2008, 40 Bonnas 2009, 41 Livnah et al. 1996, 42 Wrighton et al. 1996, 43 Kawakami et al. 2001

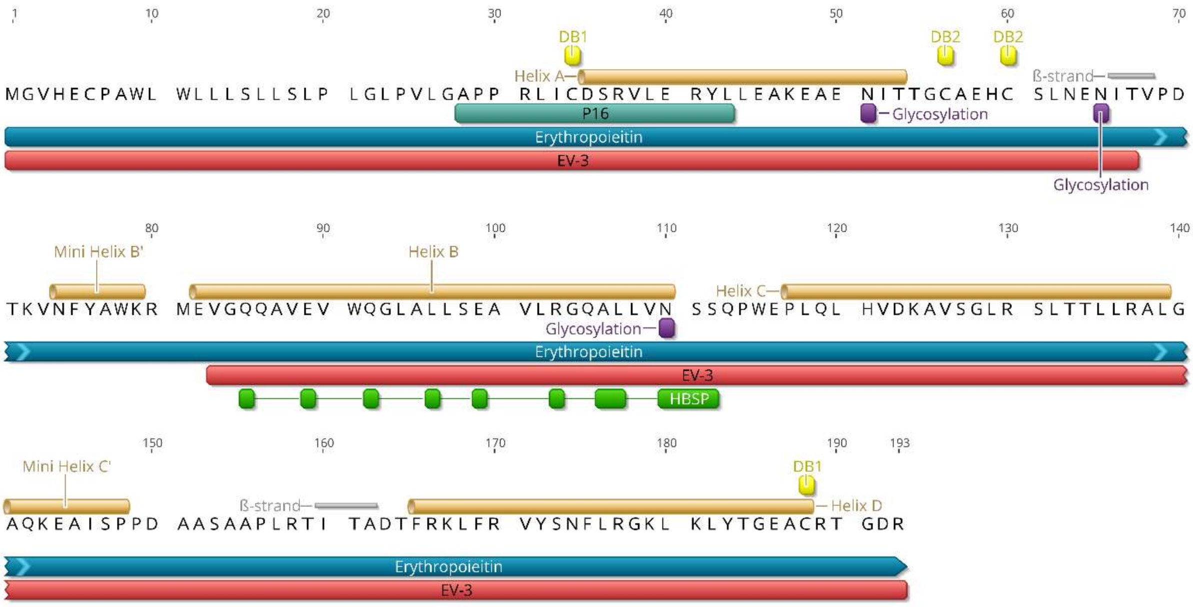

Figure S1: Graphic representation of amino acid sequences of Epo and corresponding Epo-like molecules. The Epo sequence is depicted in blue. Structural features of Epo are shown above its sequence while Epo-like molecules are shown below. Its four alpha-helices (A-D) are indicated in yellow and the β-strands in grey. Glycosylation sites are marked in purple. DB1 and DB2 indicate disulfide bonds. The Epo-mimetic peptide P16 is labeled in pale green whereas HBSP is labeled in bright green. The human splice variant EV-3 lacking exon 3 is represented in red.
